# Caffeine as an Active Ingredient in Cosmetic Preparations Against Hair Loss: A Systematic Review of Available Clinical Evidence

**DOI:** 10.3390/healthcare13040395

**Published:** 2025-02-12

**Authors:** Ewelina Szendzielorz, Radoslaw Spiewak

**Affiliations:** Department of Experimental Dermatology and Cosmetology, Faculty of Pharmacy, Jagiellonian University Medical College, ul. Medyczna 9, 30-688 Krakow, Poland; ewelina.szendzielorz@uj.edu.pl

**Keywords:** hair loss, alopecia, effluvium, baldness, treatment, caffeine, clinical trials, systematic review

## Abstract

**Background/Objectives:** Hair loss (alopecia or effluvium) can significantly affect the self-esteem and psychosocial well-being of patients, resulting in a reduced quality of life. It may herald a systemic disease, nutritional deficiency, or side effects of pharmacotherapy. Current therapeutic options for hair loss are not always satisfactory and may be associated with considerable side effects; therefore, new solutions are still sought. Caffeine seems to be an effective agent against hair loss thanks to its stimulating effects on cell growth and good penetration into the hair follicle. The aim of this study was to systematically review published clinical trials of topical caffeine preparations against hair loss. **Methods:** We searched PubMed, Scopus, and Web of Science for clinical trials investigating the efficacy of topical caffeine products in hair loss, published until 29 November 2024. The quality of evidence was assessed using the GRADE classification. **Results:** The query returned 1121 articles, of which 9 ultimately met the inclusion criteria. In total, 684 people with androgenetic alopecia, excessive hair loss, or hair thinning were included in these trials. In all studies, conclusions were in favor of topical caffeine treatment; however, the level of scientific evidence was medium in 3 studies, low in 1, and very low in the remaining 5. Their major flaws included the lack of randomization and placebo and control groups, as well as the lack of information on the caffeine concentration in the topical products. **Conclusions:** Results from studies published to date suggest that topical caffeine preparations are safe and effective against hair loss. Nevertheless, better-designed clinical trials of well-defined caffeine products are required for an ultimate statement. Commercial hair products with caffeine offered on the market nowadays may be worth a try, but due to incomplete scientific data and product information, satisfactory outcomes are not guaranteed.

## 1. Introduction

Hair loss, referred to as alopecia or effluvium, is a condition frequently encountered by healthcare providers, as the rates of people suffering from excessive hair loss are on the increase [1]. More than 80 million people are affected by hair loss in the USA alone [2]. It may not be a life-threatening condition, but it causes a considerable psychosocial burden [3]. Patients with baldness have a higher incidence of depression, anxiety, and social phobia than the general population [4]. People suffering from hair loss not only worry about losing their physical attractiveness but also experience a decrease in social attractiveness [5]. Androgenetic alopecia (AGA) has a considerable psychosocial impact on both men and women, with women experiencing greater anxiety due to the severe course of alopecia [6]. Hair loss is also among the relevant factors lowering the quality of life of women during and after cancer therapy [7,8,9]. In the case of alopecia areata, both men and women reported emotional and psychological effects, including feelings of sadness, shame, and frustration [10]. Data from multiple studies indicate the need to address psychosocial comorbidities associated with AGA, as patients with hair loss would benefit from psychological support [11]. Moreover, hair loss or thinning may be a revealing symptom of systemic diseases [2,12]. It may signal micronutrient or macronutrient deficiencies [13,14,15]. Telogen effluvium (TE) is common in acute infections [16,17]. Studies on the course of COVID-19 showed that younger patients suffered mainly from fatigue and dysosmia, while hair loss was more common among women and elderly patients [18].

Medicines are a common cause of hair loss. The use of GLP-1 receptor antagonists is associated with higher rates of hair loss as compared with other antidiabetic medications [19]. A multicenter retrospective study showed that patients treated with anticonvulsants frequently experience hair loss and alopecia [20]. Telogen effluvium has been reported as one of the common side effects of anti-seizure medication (ASM); therefore, patients complaining of hair loss while on ASM should undergo further evaluation and a specialist consultation [21]. Oncology patients experience many adverse symptoms in the hair, skin, and nails, for which they often reach for over-the-counter (OTC) medicines, typically with little or no evidence of their effectiveness [22]. The problem of hair loss in the course of oncological treatment has a large impact on the quality of life and calls for new solutions to reduce the anxiety associated with hair loss [23]. In the Food and Drug Administration’s (FDA) Adverse Event Reporting System, the largest proportion of reported alopecia was ascribed to immunomodulators and monoclonal antibodies, followed by hair loss medications, which paradoxically may also induce hair shedding, contraceptives, and anti-TNF biologics [24]. The possibility of initial hair loss as a side effect of anti-hair loss medicines like topical minoxidil or oral finasteride seems to be inherent to their mechanisms of action, as they initially accelerate the telogen phase (hair loss) before driving hair units into the desired anagen phase (hair growth) [24]. Nevertheless, the initial hair fall may discourage some patients from using evidence-based treatments and seeking alternative methods, which illustrates why proper diagnosis and rapid treatment are so important [25,26]. Regardless of alopecia type, the cause should be established, and aimed treatment initiated as soon as possible because early treatment promises a better outcome [27]. In order to support the overtasked health specialists, deep learning (AI)-based systems are developed to facilitate an early diagnosis of hair problems and a better safety assessment of hair products [28,29].

Effective treatments for hair loss are in high demand [30]. At present, the US Food and Drug Administration (FDA) and the European Medicines Agency (EMA) only approve topical minoxidil and oral finasteride as treatment options for AGA [31]. These pharmacological treatments are effective but have their limitations, including the risk of adverse events, unsatisfactory compliance under long-term treatment, and a high relapse rate. Among the adverse effects of finasteride, decreased libido, headache, and gastrointestinal problems are cited most frequently [32]. The response to minoxidil depends on the activity of sulfotransferase in the hair follicles that converts the prodrug into the active form of minoxidil sulfate [33]. Numerous cases of allergic contact dermatitis to topical minoxidil are reported in the medical literature [1]. Moreover, in most topical medications for hair and scalp, the active ingredient is dissolved in alcohols or propylene glycol, which may cause irritation to the skin and damage the hair structure [34]. Therefore, more effective hair loss-preventing and hair growth-promoting products are urgently needed. A number of non-drug interventions against hair loss show promising results in observational studies and clinical trials [35]. Arguably, the most convenient formulation for hair problems is shampoo because its regular application fits into the everyday habits of most people, including treated persons, thus enhancing compliance. Pharmaceutical and cosmetic companies should, therefore, focus on shampoo ingredients that would stop excessive hair loss and stimulate anabolic processes in the hair follicle. The search for such ingredients should prioritize molecules that easily penetrate into the hair follicle and have confirmed beneficial effects on the hair [36]. Among the ingredients that gained a vivid interest in this regard is caffeine. The easy availability and widespread consumption of caffeine have inspired numerous studies examining its impact on human health, including cardiovascular, neurological, and metabolic functions, as well as cancer immunity [37]. In dermatology, a wide range of potential applications of caffeine have been proposed [38]. As a widely recognized active ingredient of coffee, which is a “cult” drink cherished by many, caffeine seems very attractive as a cosmetic ingredient, especially in the context of growing interest in natural cosmetics that are perceived by many as safer and more effective [39]. The molecular mechanisms underlying the effects of caffeine on hair growth also speak in favor of using it as an anti-hair loss or hair growth-promoting agent [40]. Nevertheless, in vitro studies and theoretical reasoning cannot replace empirical data from real-world clinical trials.

## 2. Aim

The aim of the present article was, therefore, to collate the published data from clinical trials on caffeine efficacy against hair loss.

## 3. Materials and Methods

### 3.1. Search Strategy

The systematic review was performed according to PRISMA and PICO (Patients, Interventions, Comparisons, Outcomes) protocols. PubMed, Scopus, and Web of Science databases were searched without time and language filters.

### 3.2. Evidence Acquisition

From 10 January 2024 to 29 November 2024, scientific publications indexed in PubMed, Scopus, and Web of Science were searched using the following query: (caffeine OR “1,3,7—trimethylpurine—2,6-dione”) AND (hair OR alopecia OR baldness OR effuvium OR pilo* OR pili). The option “all fields” was enabled, and no additional filters or publication date limits were applied. The initial search returned 1695 articles, of which 574 duplicates were identified and removed. The remaining 1121 articles were assessed by the first author by title and abstract, and articles that passed this initial screening underwent a second, full-text review by both authors independently to single out articles meeting the inclusion criteria for the in-depth analysis and data extraction. In the case of papers selected by one co-author only, the consensus was reached through a discussion.

### 3.3. Inclusion and Exclusion Criteria

Only original articles reporting human clinical trials on the effects of topical caffeine preparations on hair loss (alopecia, effluvium) or hair thinning were included. In vitro and animal studies were excluded from the analysis. Review articles, as well as original studies published only as abstracts, posters, or meeting reports, were also excluded. Priority was given to randomized controlled trials (RCTs) with caffeine preparations of a given concentration and appropriate, well-defined comparators to strengthen the quality of evidence. In addition to the study design, special attention was paid to the concentration of caffeine, mode of application, duration of treatment with caffeine-based preparations, and outcome measures. The GRADE classification was used to assess the quality of evidence for the studies included in this review. This systematic approach categorizes the quality of evidence of a study as very low, low, moderate, or high [41].

### 3.4. Data Extraction

**Figure 1:**
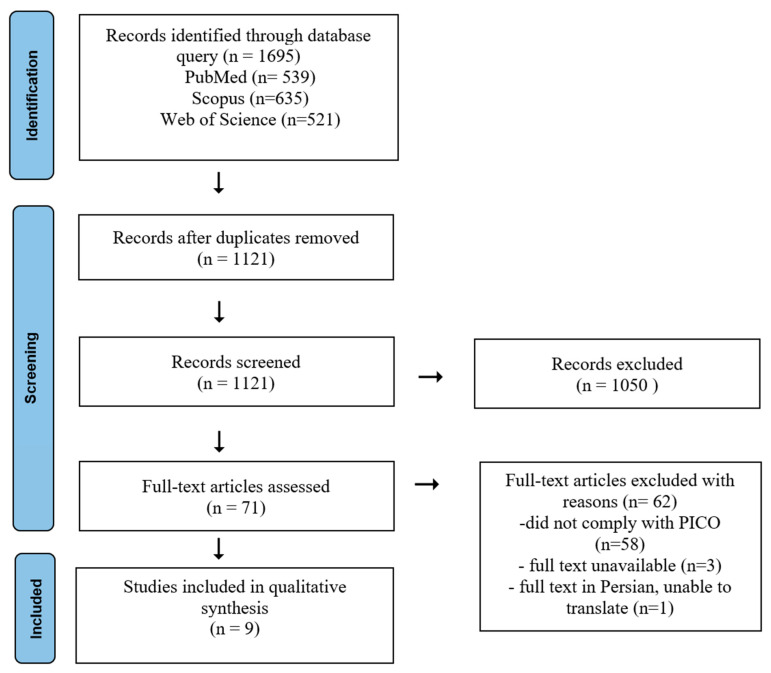
PRISMA protocol for data acquisition.

Data of relevance to the present review were extracted from the selected articles into predesigned tables reflecting the PICO criteria (Table 1) by the first author and subsequently reviewed by the second author. The final content of the tables was accepted by both co-authors. The entire search and selection process is presented in Figure 1.

## 4. Results

Out of 1121 articles identified, 9 clinical trial reports met the above-mentioned inclusion criteria [42,43,44,45,46,47,48,49,50]. Four trials studied the efficacy of caffeine applied to the scalp in the form of a shampoo (rinse-off products), while the remaining five trials involved leave-on formulations: 2 serums, 1 lotion, 1 liquid, and 1 foam. Six (67%) trials involved people with AGA, two (22%) people with excessive hair loss, and one study (11%), people with “hair thinning” (Table 2).

The total number of individuals analyzed in all articles was 684, including 388 men (56.7%), 30 women (4.4%), and 266 (38.9%) individuals of undisclosed gender; the cumulated age range was 18 to 75 years. The observation time was in the range of 2–6 months. Four studies focused on caffeine as the sole active ingredient (Table 3), while in the remaining five, complex mixtures of caffeine and other ingredients considered active were used (Table 4). The actual concentrations of caffeine in the products tested were disclosed in only 2 out of 9 analyzed studies [42,47].

Overall, patients reported good tolerability and satisfaction with caffeine products. In one study, a caffeine shampoo met the expectations of 67% of participants, who attributed increased hair strength, reduced progression of alopecia, and reduced hair loss to the shampoo. Furthermore, 85% of the participants expressed an intention to continue treatment because, in their opinion, the shampoo formulation was not only effective but also convenient to use [44]. Topical caffeine serum also resulted in high patient satisfaction with a perceived improvement of 80–100% in most aspects of hair quality, no observed side effects, and a reported compliance rate of 100% [49]. In a study of a complex lotion with Procapil™ and caffeine, 84.2% of participants rated their hair loss as “improved” or “very improved”, while no side effects such as dryness, itching, redness, flaking, folliculitis, or burning sensation were reported [50]. In a study of a serum containing 30 ingredients, including caffeine, all participants expressed their satisfaction with improved hair growth, hair thickness, and hair strength, as well as reduced hair loss, while nobody complained of any irritant reactions such as redness, dryness, itching, or a burning sensation on the scalp [48]. In a study of shampoo with caffeine and adenosine, 71% of participants expressed overall satisfaction with the treatment as compared to 66% in the control group that used an identical-looking shampoo without the active agents; the authors also reported that “some participants” complained of dry hair [47]. Increased scalp oiliness during the treatment with a caffeine mousse was reported by 1, and scalp flushing by 2 out of 29 participants in another study [46]. Finally, 1 of 79 controls using a 5% minoxidil solution reported a headache, while none of the 82 participants using a 0.2% caffeine solution complained of any adverse events [42]. No other adverse events were reported in the trials covered by the present review.

## 5. Discussion

The World Health Organization (WHO) defines the quality of life as a subjective assessment of the perception of reality through the prism of culture and personal values, which explains why in societies that highly regard a person’s physical appearance, excessive hair loss is perceived as a problem more serious than other, oftentimes more severe and debilitating, diseases [51]. The difficult and lengthy process of treating chronic ailments requires clear guidelines that take into account the patient’s values and preferences [52]. Among the key values determining the quality of life during long-term treatment is the patient’s need not to “appear ill” in the presence of loved ones (e.g., children and grandchildren), which, among other aspects, includes the status of the hair [53]. Therefore, tackling the problem of hair loss contributes to improving the overall quality of life regardless of the underlying disease. The number of anti-hair loss products on the market is constantly growing, and likewise, the number of their ingredients with claimed beneficial effects on the hair is growing. An analysis of 92 anti-hair loss products (shampoos, lotions, serums, and conditioners) sold in Poland from 2018 to 2019 revealed that among 448 unique ingredients that were declared in the product information, 207 were advertised as “active,” although scientific evidence was available only for 8 of them (4%), including caffeine which was present in 13 (14%) products [54]. Worryingly, some products contained substances with known adverse effects on hair growth, e.g., 28 (30%) “anti-hair loss products” contained niacinamide, which, according to the available scientific evidence, induces hair loss rather than growth [55,56]. Another study of anti-hair loss shampoos available on the Polish market from 2022 to 2024 revealed 39 “trichological shampoos” (a marketing term used for products dedicated to hair problems) with 112 unique ingredients advertised as “active against hair loss”. Again, for the vast majority of these ingredients, there was no scientific proof whatsoever of any beneficial effects on the hair. Caffeine, one of the few ingredients with a substantial body of evidence for the actual effectiveness, was listed as the ingredient of five (13%) such “trichological shampoos” [57].

According to the current understanding, the main beneficial effects of caffeine in the hair unit are due to the molecule’s interaction with the adenosine pathway, which causes an increase in cAMP levels, which results in the stimulation of metabolic activity in the hair follicle; moreover, caffeine possesses antioxidant properties which prevent degenerative processes in the cells [58]. It appears that the effects of caffeine on the hair are studied more extensively than most other active components; e.g., in a previous systematic review, we could only find three published clinical trials of placenta derivatives in hair loss [59]. Nevertheless, the majority of caffeine research is based on in vitro or animal models, which are quite distant from the real-world situations of everyday use of a topical product on the human scalp. As shown in this systematic review, there are a handful of clinical trials carried out in humans, but the overall quality of most of them is unsatisfactory due to numerous flaws in their design. The majority of the nine published clinical trials on topical caffeine are open-label, uncontrolled studies. Only two of them were randomized, double-blind trials with appropriate controls, among which the concentration of caffeine was disclosed in only one (for more details, see Tables S1 and S2 in the Supplementary Materials). Moreover, the majority of the trials of caffeine products were performed in men with AGA (67% of all participants), and only one trial studied women with telogen effluvium (TE). One study combined people with TE and AGA in one group, and another two used poorly defined inclusion criteria of “hair loss” or “thinning hair”.

Like any intervention studies, trials of topical caffeine in hair loss are susceptible to selection bias, i.e., a situation where a flawed selection leads to systematic differences between compared groups (treatment, placebo arm) regardless of the interventions themselves. When looking at Table 3, a stark gender imbalance becomes apparent, with only 30 women (4.4%) included altogether in the studies. This may lead to a considerable bias, which limits the credibility of the conclusions about possible beneficial effects of caffeine on the hair. Gender bias due to male or female underrepresentation is known in various areas of clinical research, and institutional efforts are undertaken to overcome this obstacle [60,61,62,63,64]. In future studies of caffeine in alopecia types that affect both genders, e.g., drug-, nutritional deficiency-, or infection-induced hair loss, the recruited study groups should be representative of the affected populations, including the gender balance. This postulate does not apply to studies of gender-specific hair loss, e.g., female pattern hair loss (FPHL) and male pattern hair loss (MPHL), which should be tested in respective gender groups.

Humans, both study participants and personnel, are susceptible to suggestion. Typically, non-blinded investigators tend to overestimate the effects of an intervention by 7–43% [65]. Therefore, future studies of caffeine in hair loss should be double-blind, where neither the participants nor the investigators know whether a given person was treated with a placebo or verum. The inclusion of a placebo arm in future clinical trials of hair products is especially crucial due to seasonal fluctuations in spontaneous hair shedding observed in humans. In Europe, the rates of hair shedding and telogen hair are highest in late summer and lowest in early spring, a trend documented independently in the UK, France, and Switzerland [66,67,68]. In the USA, the same trends in hair loss are reflected by the volume of Google searches [69]. This perennial cycle may be a relevant confounding factor in the trials of hair loss. For example, in an uncontrolled trial started in autumn and concluded in spring, the observed decrease in hair loss might falsely hint at the effectiveness of a de facto ineffective intervention due to coincidence with the seasonal trends. The most effective method to minimize this type of bias is the randomization of recruited participants by randomly assigning them to either the investigated intervention (verum) or sham treatment (placebo) [70]. The utilization of a placebo arm would help to expose such situations, even though researchers have been long aware that there is more to a placebo than just a non-effective treatment [71]. Positive effects observed in considerable subgroups of placebo-treated patients have been attributed to increased healthcare support, more frequent clinical assessments, positive expectations, and improved adherence to concomitant medication which altogether contribute to the observed effects of placebo [72]. Also, a reverse phenomenon, the nocebo effect, should be taken into account when analyzing clinical data [73,74].

In addition to the efficacy of the product itself, other important factors that determine the overall efficacy of an intervention are tolerability (lack of adverse effects), ease of application, and overall assessment of “consumer experience”, which all contribute to patient compliance and adherence to treatment [75,76]. The terms “compliance” and “adherence” are frequently considered synonyms as both refer to applying a treatment exactly as instructed; however, some authors argue that the first term suggests a rather passive attitude while the latter implies more engagement from the patient [77]. There are numerous options for measuring adherence, ranging from diaries and questionnaires filled in by study participants through monitoring the consumption of the product tested (e.g., counting pills or weighing the remaining product) to measuring metabolite levels in the blood or exposed organs [78]. The frequency of use and consumed volume of the tested products could be monitored in real time with the help of medication adherence monitoring systems [79,80]. A range of validated questionnaire tools are available for measuring adherence to topical treatments, e.g., ECOB, Morisky Medication Adherence Scale-8 (MMAS-8), Questionnaire for Adherence to TOPical treatment (QATOP), and Topical Therapy and Adherence Questionnaire (TTAQ), as reviewed recently elsewhere [81].

From a methodological point of view, the patient’s subjective evaluation of the product may seem a “weak” outcome measure, but it has a strong influence on the patient’s decision whether to adhere to the treatment regimen or not [82]. Patient-reported outcomes measures (PROMs) are increasingly used in clinical trials to provide patients’ perspectives regarding the health-related quality of life and satisfaction with the treatment. They capture patients’ daily experiences with their treatment that cannot be measured in any other way [83]. Knowing and understanding the patients’ experience is crucial for the selection of the most effective interventions. Therefore, the use of PROMs in clinical research is promoted by scientific consortia and recommended by regulators, including the FDA and EMA [84]. The main obstacles to a broader implementation of PROMs in dermatology include costs, difficulty in selecting the right PROMs for a given disease, and a lack of understanding of the added value of PROMs among researchers. As a result, PROMs are sometimes considered an excessive burden to study participants, which may reduce their motivation to answer all the questions, thus potentially harming the data completeness and quality [85,86].

Taking into account that patient satisfaction measures are subjective and prone to bias, objective measures should also be mandatory in future trials. Traditional methods of hair loss and growth quantification include the daily hair count, standardized wash test, 60 s hair count, hair weight, contrasting felt examination, and the trichogram, i.e., an assessment of the percentage of anagen and telogen hairs among hair samples pulled from the patient’s scalp [87,88,89,90]. Scalp biopsy with both vertical and horizontal sections may also be a source of valuable data regarding the hair unit density and status, the present phase of the hair cycle they are in, as well as a wide range of further molecular clues when combined with appropriate immunostaining [91,92,93,94]. Biomarkers of the hair status could be assessed either in biopsies of the scalp or in hair follicle dermal papilla cells (HFDPCs) retrieved with the use of non-invasive techniques [95]. Among promising candidate biomarkers of hair growth, the upregulation of alkaline phosphatase (ALP) in HFDPCs seems to stand out, a crucial enzyme in the transition of hair follicles from telogen to anagen phase [96]. Other measurable anagen-inducing factors include IGF-1, VEGF, β-catenin, versican, noggin, or WNT ligands [97,98,99,100,101,102]. On the other hand, a decrease in factors inhibiting hair growth, e.g., DKK-1 or TGF-β1, could also serve as an objective measure in future trials [96,102]. Integrated multi-omics biomarkers that combine genomic, transcriptomic, and proteomic signatures have also been proposed for monitoring androgenetic alopecia and alopecia areata [103].

It seems that the most valuable means of quantitating hair loss and hair growth nowadays are global scalp photography and phototrichogram techniques supplemented with automated digital image analysis based on machine learning, deep neural networks, or other rapidly developing AI incarnations [104,105,106,107,108,109,110,111]. It seems that these non-invasive, high-throughput methods may offer reproducible, objective measures that would allow for a fast and reliable assessment of caffeine efficacy in large groups of patients, which could significantly improve and strengthen the conclusions from future clinical trials.

The actual concentration of caffeine in the products tested was disclosed in only 2 (22%) out of 9 studies included in the present review [42,47]. This figure seems strikingly low when compared to studies on the ergogenic effects of caffeine, where the doses of caffeine were reported in 68% of studies [112]. The lack of clinical dose-response data is a significant obstacle in assessing the efficacy of caffeine against hair loss. Reporting precise caffeine concentrations in future trials would allow for reliable dose-response analysis, as both beneficial and detrimental effects have been ascribed to caffeine, a bioactive molecule that may follow a hormetic dose-response relationship. In a hormetic relationship, low doses of a substance have beneficial (stimulatory) effects, while higher doses have detrimental (inhibitory or toxic) effects on a system [113]. In the founding paper on the theory of hormesis [114], caffeine was mentioned as a model substance following the hormetic pattern, which was partly confirmed in later experimental research [115,116,117]. The present data are insufficient to assess at what concentration range caffeine would be most beneficial for the hair and whether there exists a threshold for possible detrimental effects. Moreover, these questions should be answered separately for the rinse-off (shampoos) and leave-on (e.g., serums) products.

While preparing this systematic review, we encountered an unexpected limitation to the available body of evidence. Our bibliographic query revealed two papers that seemed relevant to the topic but could not be included in the systematic review for rather prosaic reasons. One article was written in Persian. According to the English-language abstract, the authors compared the effects of minoxidil 2.5% versus a mixture of minoxidil 2.5% with caffeine at an undisclosed concentration in 60 patients with androgenic alopecia (gender and age not disclosed) over a period of 150 days [118]. The authors concluded that the combination with caffeine was “significantly better”; however, the abstract lacked details sufficient for an analysis of this work’s methodology and results. An attempt at automatic translation from Persian produced a text that obviously could not be trusted (e.g., confusing syntax, reversal of numerical values). In a final bid, we individually contacted three co-authors of the work via email or ResearchGate profiles, asking for insight into the necessary data, but we received no response. The second article was published in English; however, the PDF version available from the publisher’s site was lacking pages (170–171) with the crucial data on the trial methodology necessary to assess the GRADE level of evidence [44]. We contacted the publisher, who replied that this was the only scanned version they had. Again, we individually contacted and asked for help from two co-authors of the paper, to no avail. Based on the available part of the article, 30 males with androgenic alopecia, 19–55 y.o., were treated with a caffeine lotion for 4 months. Neither caffeine concentration nor a control group was mentioned in the available part. The authors reported a “reduction in hair loss” in 83% of the participants, a “reduction in premature hair loss” in 43%, as well as an “improvement in hair texture” (“force, tensile strength”) in 53% of participants. The pull test was mentioned among outcome measures, along with the participant’s and investigator’s assessments; however, we could not analyze the study in sufficient detail due to missing pages with the description of the methodology. Therefore, these two papers could not be included in the present systematic review.

## 6. Conclusions

Hair loss may herald systemic diseases, nutritional deficiency, or side effects of pharmacotherapy. Regardless of the underlying mechanism, the emergence of alopecia or effluvium is a stressful event that significantly affects the patient’s quality of life. The regulator-approved therapeutic options include minoxidil and finasteride; however, proven effectiveness goes hand in hand with the risk of untoward effects. Therefore, there is a great demand for new active substances that could replace or complement the established treatments. Caffeine is one of such promising molecules. Collated data available from the published clinical trials altogether speak in favor of caffeine as an anti-hair loss agent in androgenic alopecia and, to a lesser degree, also in telogen effluvium. Caffeine-containing hair products seem generally well-tolerated and accepted by users. This provisional assessment, however, has to be taken with caution due to the low quality of most trials published to date, as well as the lack of dose-response analyses. Good quality, randomized, long-term clinical trials with a matched comparator (preferably identical hair product with less caffeine), as well as dose-response studies of caffeine in hair loss, are necessary for dispelling doubts regarding this treatment modality. Due to the lack of respective trials, no advice can be given with regard to the use of topical caffeine in other types of hair loss. In collating the above, practical advice for the time being would be that commercial anti-hair loss products with caffeine offered on the market may be worth a try, but with undisclosed caffeine content, satisfactory outcomes are not warranted.

## 7. Future Perspective

In a recent systematic review of the placenta and its derivatives in hair loss, we proposed a list of postulates which should be addressed in future clinical trials [61]. Based on the present systematic review, we suggest that future clinical trials of topical caffeine products in hair loss use the following:

- Are conducted in a double-blind, randomized manner, where the caffeine preparation (verum) is compared with a matched placebo, preferably an identical hair product devoid only of caffeine;
- Are conducted in sufficiently large groups of participants, representative of all patients with a given type of hair loss with regard to symptoms, severity, age, and gender balance;
- Test well-defined test products with all ingredients and concentrations mentioned, especially those of caffeine. Comparisons of different caffeine concentrations within one trial would be an important step forward;
- Ensure that control groups are matched to the verum groups in all the above-mentioned criteria to avoid selection bias;
- Last sufficiently long to detect changes in the observed physiological and pathological processes, with the known seasonal variation in hair growth and loss taken into account;
- Involve objective measures of the effect, like the number of shed hairs, rates of anagen and telogen hairs, number of hairs per area unit, hair thickness, and velocity of growth, measured with the use of reproducible, validated, and standardized methods;
- Involve patient-reported outcome measures, e.g., the influence of the treatment on the quality of life or willingness to continue the treatment;
- Systematically monitor the participants’ adherence to the treatment protocol;
- Systematically monitor all adverse events that emerge in the course of the study.

## Figures and Tables

**Table 1:** Inclusion criteria (PICO) for the human studies included in the present systematic review.

PICO Criteria	Description
Patients	People suffering from baldness, hair loss, effluvium, alopecia, hair thinning
Intervention	Caffeine in topical anti-hair loss preparation
Comparator/Control	Placebo, other topical anti-hair loss preparations, no control
Outcomes	Trichogram results, trichoscopy results, number of hairs shed, evaluator assessment, patient assessment

**Table 2:** Types of hair problems covered by clinical trials included in the present systematic review.

Hair Problem	Main Features	Diagnostic Methods	Number of Participants	Refs.
Androgenetic alopecia	Miniaturization of hair follicles in androgen-dependent areas	Dermatoscopy, Trichoscopy	538	[42,43,44,46,49,50]
Telogen effluvium	Occurs about 3 months after triggering incident (e.g., infection, drug use, hormonal disorders, metabolic diseases, nutritional deficiencies, stress)	Pull test, trichogram, laboratory tests for underlying conditions	62	[45,48]
“Hair thinning”	Noticeable reduction in hair density	Dermatoscopy, Trichoscopy	84	[47]

**Table 3:** An overview of clinical trials on the efficacy of caffeine preparations against hair loss (see Supplementary Materials, Table S1 for more details).

Study Design	Participants	Intervention	Control	Outcome	GRADE	Ref.
Prospective RCT, open-label	210 M with AGA	2 mL of a 0.2% caffeine solution (leave-on)	1 mL of a 5% minoxidil solution	Effects of 0.2% caffeine comparable to 5% minoxidil	Medium	[42]
Prospective RCT, double-blind	66 M with AGA	7 mL of shampoo with caffeine (rinse-off)	7 mL of the same shampoo less caffeine	Objective and subjective improvement greater after caffeine than placebo	Low	[43]
Prospective, uncontrolled, open-label	30 M with AGA	7 mL of shampoo with caffeine (rinse-off)	None	Objective and subjective improvement	Very low	[44]
Prospective, uncontrolled, open-label	30 F with TE	Shampoo with caffeine (rinse off)	None	Objective and subjective improvement	Very low	[45]

Abbreviations: AGA—androgenic alopecia; TE—telogen effluvium; F—female(s); M—male(s); mo—month(s); RCT—randomized controlled trial; GRADE—level of evidence assessed in line with the Grading of Recommendations Assessment, Development, and Evaluation (GRADE) system of rating quality of evidence [41].

**Table 4:** An overview of clinical trials on the efficacy of combined preparations with caffeine against hair loss (see Supplementary Materials, Table S2 for more details).

Study Design	Participants	Intervention	Control	Outcome	GRADE	Ref.
Prospective, RCT, double-blind	62 M with AGA	Foam with 10 “active” ingredients including caffeine	Foam without 10 “active” ingredients	Foam with caffeine et al. better than vehicle foam	Medium	[46]
Prospective, RCT, single-blind	84 healthy F and M with self-perceived “thinning hair”	Shampoo with 0.4% caffeine and 0.2% adenosine	Same shampoo without caffeine and adenosine	Improvement after shampoo with caffeine and adenosine, but not after control shampoo	Medium	[47]
Prospective, uncontrolled, open-label	32 F and M with hair loss	Serum with a complex mixture of 30 ingredients, including caffeine	None	Improvement of hair condition and growth	Very low	[48]
Prospective, uncontrolled, open-label	150 M and F with AGA	Serum in a roller with a complex mixture including caffeine	None	Improvement of hair parameters in crown and vertex, but not frontal area	Very low	[49]
Prospective, uncontrolled, open-label	20 M with AGA	Topical formulation containing caffeine, 3% Procapil™ and zinc PCA	None	Improvement in hair growth, decrease in hair loss	Very low	[50]

Abbreviations: AGA—androgenic alopecia; TE—telogen effluvium; F—female(s); M—male(s); mo—month(s); RCT—randomized controlled trial; GRADE—level of evidence assessed in line with the Grading of Recommendations Assessment, Development, and Evaluation (GRADE) system of rating quality of evidence [41].

## Data Availability

The original contributions presented in the study are included in the article/Supplementary Materials; further inquiries can be directed to the corresponding author.

## Supplementary Materials

The following supporting information can be downloaded at https://www.mdpi.com/article/10.3390/healthcare13040395/s1; Table S1: Detailed results of clinical studies on the efficacy of caffeine preparations in hair loss; Table S2: An overview of clinical studies on the efficacy of combined preparations with caffeine in hair loss. Ref. [119] was cited in Supplementary Materials.

## Abbreviations

The following abbreviations are used in this manuscript:

AGA	Androgenic alopecia
EMA	European Medicines Agency
F	Female
FDA	Food and Drug Administration
GRADE	Grading of Recommendations Assessment, Development, and Evaluation
M	Male
OTC	Over-the-counter medicines
TE	Telogen effluvium
WHO	World Health Organization
