# Supplementary material for: Caffeine as an Active Ingredient in Cosmetic Preparations Against Hair Loss: A Systematic Review of Available Clinical Evidence
Source: Healthcare (Basel). 2025 Feb 12;13(4):395. doi: 10.3390/healthcare13040395 (PMC11855793; doi:10.3390/healthcare13040395)
Supplement: Supplementary file 1 [file healthcare-13-00395-s001.zip › healthcare-3410952-supplementary.pdf]

Supplementary material to the article
"Caffeine as an Active Ingredient in Cosmetic Preparations Against Hair Loss: A Systematic Review of Available Clinical Evidence"

Table S1. Detailed results of clinical studies on the efficacy of caffeine preparations in hair loss.

Study Design	Caffeine Preparation and Application	Comparator Preparation and Application	Inclusion Criteria	Number of Patients, Gender	Duration of Treatment	Duration of Follow-Up	Outcome Measures	Main Reported Outcomes	Authors' Conclusion in the Article	Reviewers' Summary	Remarks/ Factors Influencing Level of Evidence	Evidence Level (GRADE)	Ref.
Multicenter, prospective, randomized, active-controlled, open-label, noninferiority study	I: 2 ml of a 0.2% caffeine-solution applied on the scalp twice a day (leave-on)	C: 1 ml of 5% minoxidil solution applied on the scalp twice a day (leave-on)	M with AGA, Hamilton-Norwood stage III–V, ≥20% telogen hairs on trichogram	210 M recruited, aged 18-55, 82 P compl. the I arm, 79 P compl. the C arm	6 mo	6 mo	IA: anagen rate on trichogram and changes in anagen rate	Mean impr. in anagen rate I: 10.59%; C: 11.68% (difference: 1.09%, ns).	"A caffeine-based topical liquid should be considered as not inferior to minoxidil 5% solution in men with androgenetic alopecia."	Effects of 0,2% caffeine comparable to s 5% minoxidil	Lack of placebo group	Medium	[1]

Randomi-zed, controlled, double-blind, parallel group study	I : 7 ml of shampoo with caffeine (undiscl. conc.) applied once a day, kept on the scalp for 2 min (rinse-off)	C : 7 ml of the same shampoo less caffeine, the same application protocol	M with AGA, Hamilton-Norwood stage II-IV; ≥18 hairs on pull test	66 M, aged 23-55, 33 P in I arm , 33 P in C arm	6 mo	6 mo	PA and IA quest.	Subjective intensity of hair loss decreased more in the I than C group ($p=0.002$). PA: "normalization of hair loss" more pronounced in the I than C group ($p<0.001$), "amount of hair in the basin" decreased more in the VI than C group ($p<0.002$), "strength and thickness of hair" impr. more in the I than PC group ($p<0.001$). More participants in I (84.8%) than C (36.4%) group wanted to continue the treatment ($p<0.001$). IA: "hair strength and thickness" impr. more in the I than C group ($p<0.001$), "balding" impr. more in the I than C group ($p<0.001$), "falling out of hair" impr. more in the I than C group ($p<0.002$), Investigator's overall preferrence of I (72.7%) higher than C (33.3%, $p=0.003$). Patient satisfaction rate with I greater than C (84.8% vs 36.4%, $p<0.001$)	"Our data support the beneficial effects of topical application of caffeine in AGA"	Impr. in subjective measures during the treatment significantly bigger after caffeine treatment than placebo.	No information on caffeine concentration, arbitrary scales for all measures, no pull test results reported, sponsored by producer of tested lotion	Low	[2]

Prospective, uncontro-lled, open-label	7 ml of a shampoo with caffeine (undiscl. conc.) was applied on hair and scalp once a day, and kept for 2 min. (rinse-off)	None	M with AGA, Hamilton-Norwood stage II-IV; ≥15 hairs on pull test	30 M recruited, aged 18–55	6 mo	6 mo	Pull test, PA and IA quest.	Pull test: the mean number of pulled hair decreased from 20.07 at start down to 18,63 after 3 months (-7.5%) and 17.43 (-13.1%) after 6 months, both p<0.001) Increased "strength of hair", decreased "progression of balding", decreasing "extent of falling out hair" and decreased "intensity of hair loss" (self-assessment on an arbitrary scale, each p<0.001) 67% PA satisfied with the product	"Finally, we can say that caffeine is a promising substance for the cosmetic treatment of male AGA, and its formulation as a shampoo is effective as well as comfortable for male subjects, who prefer a shampoo to a lotion for daily use"	Significant impr. in objective and subjective measures during the treatment; not clear how much of it attributable to caffeine	Lack of placebo or comparator substance, no information on caffeine concentratio n, arbitrary scales for most measures, sponsored by producer of tested shampoo	Very low	[3]
Prospective, uncontro-lled, open-label	I: shampoo with caffeine (undiscl. conc.) was applied on hair and scalp once a day, and kept for 2 min. (rinse-off).	None	F with TE, "copious" hair loss that begun in preceding 2 mo	30 F, aged 18-40	6 mo	3 and 6 mo	Pull-test , IA quest. after 3 and 6 mo of treatment.	Pull test: the mean number of pulled hair decreased from 21.2 at start down to 20.3 after 3 months (-4.2%, ns) and 19.2 (-9.4%) after 6 months (p=0.003) PA: impr. "intensity of hair loss" (p<0.001), decreased "number of hairs during daily combing" (p<0.001), impr. "strength of hair" (p<0.001), impr. "tension/dryness" (p=0.016). IA: impr. "strength of the hair" (p=0.002), decreased "extent of the out falling hairs" (p<0.001), decreased "progression of balding" (p=0.001), impr. "scaling/dandruff" (p=0.016).	"(…) the product has been shown to have a good cosmetic efficacy in the treatment of female TE."	Impr. in objective and subjective measures during the treatment; not clear how much of this was attributable to caffeine	Lack of placebo or comparator substance(T E is self-limiting in many cases), no information on caffeine concentratio n, arbitrary scales for most measures	Very low	[4]

Abrreviations: AGA – androgenic alopecia; TE – telogen effluvium; conc. - concentration; I – intervention (verum, test treatment); incr. – increase(d); decr. – decrease(d); impr. – improved/improvement; C – comparator (placebo, sham treatment); compl. – completed; P – participant(s); M – male(s); F – female(s); PA – participant's assessment; IA – investigator's assessment; quest. – questionnaire; ns – non significant; nass – no assessment of statistical significance reported; mo – month(s); w – week(s); GRADE – level of evidence assessed in line with the Grading of Recommendations Assessment, Development, and Evaluation (GRADE) system of rating quality of evidence [41].

References for Table S1:

1. Dhurat, R.; Chitallia, J.; May, T.W.;Ammani M,; Madhukara, J.J.; Anandan, S.; Vaidya, P.; Klenk, A. An Open-Label Randomized Multicenter Study Assessing the Noninferiority of a Caffeine-Based Topical Liquid 0.2% versus Minoxidil 5% Solution in Male Androgenetic Alopecia. *Skin Pharmacol. Physiol.* **2017**, *30*, 298-305. https://doi.org/10.1159/000481141
2. Sisto, T.; Bussoletti, C.; Celleno, L. Efficacy of a Cosmetic Caffeine Shampoo in Androgenetic Alopecia management. II Note. *J. Appl. Cosmetol*. **2013**, *31*, 57-66
3. Bussoletti, C; Mastropietro, F.; Tolaini, M.V; Celleno, L. Use of a Caffeine Shampoo for the Treatment of Male Androgenetic Alopecia. *J. Appl. Cosmetol.* **2010**, 28, 153-162.
4. Sisto, T.; Bussoletti, C.; Celleno, L. Role of a Caffeine Shampoo in Cosmetic Management of Telogen Effluvium. *J. Appl. Cosmetol*. **2013**, 31, 139-145.

Table S2. An overview of clinical studies on the efficacy of combined preparations with caffeine in hair loss.

Study Design	Intervention: Caffeine Preparation and Application	Comparator Preparation and Application	Inclusion Criteria	Participants	Duration of Treatment	Duration of Follow-Up	Outcome Measures	Reported Outcomes	Authors' Conclusion in the Original Article	Reviewers' Summary	Remarks/ Factors Influencing Level of Evidence	Level of Evidence GRADE	Ref.
Prospective, singe center, randomized, double-blind, vehicle-controlled study	I: foam with a composition of 10 "active" ingredients including caffeine (undiscl. conc.). 1 g of product applied twice daily (leave-on)	C: foam vehicle not containing the composition of 10 "active" ingredients. Same mode of application.	M with AGA, Hamilton - Norwood stage 2–7; telogen rate >20%.	62 M recruited, aged 19-67, 29 P compl. the I arm, 30 P compl. the C arm	6 mo	6 mo	Phototrichogram. IA (quest.) and PA (quest.)	Reduction of telogen rate in the I group greater than in C group (p = 0.02). No significant differences between I and C groups on IA and PA.	"The study demonstrated a reduction of the telogen rate by a cosmetic foam in men affected by AGA"	The test foam (complex of 10 ingredients including caffeine) significantly reduced telogen rate in men with AGA compared to the vehicle foam after 6 months.	No information on conc. of caffeine and other "active" ingredients. Not clear how much of this effect is attributable to caffeine. Single center study.	Medium	[1]
Randomized, controlled, single-blind	I: Shampoo with 0.4% caffeine and 0.2% adenosine. 10 g of product used three times a week (rinse-off)	C: Same shampoo without caffeine and adenosine. Same mode of application.	Healthy F and M with self-perceived "thinning hair", Ludwig grade I-1, I-2 or I-3 (F), Norwood Type 2, 2A, 3 or 3A (M), age >18 years.	84 P aged 20-60 years enrolled, 77 P compl. the study	3 mo	3 mo	Hair density photographs, dermoscopic hair analysis system, hair loss count at combing, hair diameter micrometry, satisfaction scores on PA (quest.)	Mean hair density in I group incr. from 118.25 to 130.03 hairs/cm² (up 9.96%, p<0.001), "no significant changes" in C group (data not shown). Mean hair loss at combing decr. in I group from 27.19 to 17.53 (down 35.5%, p<0.001), and in C group from 24.97 to 19.94 (down 20.1%, ns)	"The shampoo containing 0.4% caffeine and 0.2% adenosine exhibited efficacy in regard to reducing hair loss and for hair density enhancement after 3 months compared to the baseline."	Incr. hair density and reduced hair loss after shampoo with caffeine and adenosine. No such effect after sham shampoo.	Not clear how much of the effect is attributable to caffeine. Study not blinded on investigators' side. Single center study.	Medium	[2]

Open-label, single-center, prospective, single-arm, non-comparative study	I: serum with a complex mixture of 30 ingredients, including caffeine at position 23 (undiscl. conc.). 0,5 ml product was applied on the scalp once daily (leave-on)	None	F and M with hair loss (alopecia) ,	32 P enrolled, 29 P compl.: 15 F and 14 M aged 18-45 years	60 days	60 days	Pull test, phototrichogram, PA and IA (quest.)	Hair growth rate incr. by 32% ($p<0.01$) Hair thickness incr. by 34% ($p<0.01$) Hair density incr. by 40% ($p<0.01$) Anagen hair incr. from 57% to 80% (nass) Telogen hair decr. from 43% to 20% (nass) "Up to 42% reduction in hair fall" (nass) Decr. in scalp itchiness ($p<0.01$), redness ($p<0.01$), roughness ($p<0.01$), scaliness ($p<0.01$), and dryness ($p=0.02$).	"(…) after 60 days of use, the hair serum demonstrated effectiveness and good tolerance in improving hair growth, thickness, and strength while reducing hair loss"	Impr. of hair condition and growth after treatment with a complex product including caffeine.	Lack of randomization and control, no information on conc. of caffeine and other "active" ingredients in the products. Not clear how much of this is attributable to caffeine. Single center study. Research funded by producer of the commercial product tested.	Very low	[3]

Single center, open-label, prospective, observational, real-world study	I: serum in a roller with a complex mixture of 24 (M) or 27 (F) ingredients, including caffeine at position 6 (undiscl. conc.). Roller applied twice per day (leave-on)	None	F and M with mild to severe AGA, 18-75 y.o.	150 P enrolled (90 F, 60 M), all compl. the study.	8 w	8 w	Photography, P-filled quest. Phototrichogram in 7 P.	Hair thickness incr. in crown (17%, $p<0.05$), vertex (15%, $p<0.05$) and frontal (10%, ns) areas. Hair density incr. in crown (24%, $p<0.05$), vertex (12%, $p<0.05$) and frontal (2%, ns). PA: Decr. in hair shedding declared by 100% P, incr. frequency of new hair (90%), incr. in hair growth, hair volume, scalp coverage and amount of hair regrowth (each 80%).	"we show that twice-daily use of the novel topical serum (...) led to meaningful improvements in hair growth with high patient satisfaction and no side effects."	Significant impr. in hair parameters in crown and vertex, but not frontal area. High satisfaction rates.	Lack of randomization, no comparator, short follow-up, small group on trichogram (7 P). No information on conc. of caffeine and other ”active” ingredients in the products. Not clear how much of this is attributable to caffeine. Single center study. Research funded by producer of the commercial product tested.	Very low	[4]

Single center, open-label, prospective study	I: liquid with a complex mixture of Procapil™ 3%*, caffeine (undiscl. conc.), and zinc PCA (undiscl. conc.). Undiscl. amount of product was applied on the scalp once daily (leave-on)	None	M with AGA, Hamilton - Norwood stage II–IV, age range: 18 –57 y.o.	20 P enrolled (all M), 19 compl. the study	12 w	12 w	Photography, combing test, trichoscopic assessment, P satisfaction	IA: "impr. hair growth" by 68.5% and 68.4% after 6 and 12 w. Decr. in hair loss by 26.9% in combing test after 12 w (p=0.026). Terminal/vellus hair ratio incr. by 53% after 12 w (p=0.028). Incr. in the anagen/telogen ratio by 12.8% after 12 w (ns). No change in total hair count and density after 6 and 12 w (ns). PA: "impr. hair" reported by 12.8% after 6 and 84.2% after 12 w.	"this study found a 12-week treatment course with a topical formulation containing caffeine and Procapil 3% a beneficial and promising treatment for AGA"	Improvement in hair growth, decrease in hair loss, incr. terminal hair. High satisfaction rates.	Lack of randomization and control, no information on conc. of caffeine and other "active" ingredients in the products. Not clear how much of this is attributable to caffeine. Single center study. Research funded by producer of the commercial product tested.	Very low	[5]

Abbreviations: AGA – androgenic alopecia; TE – telogen effluvium; conc. - concentration; I – intervention (verum, test treatment); incr. – increase(d); decr. – decrease(d); impr. – improved/improvement; C – comparator (placebo, sham treatment); compl. – completed; P – participant(s); M – male(s); F – female(s); PA – participant's assessment; IA – investigator's assessment; quest. – questionnaire; ns – non significant; nass – no assessment of statistical significance reported; mo – month(s); w – week(s); undiscl. – undisclosed; PCA – pyrrolidonecarboxylic acid; GRADE – level of evidence assessed in line with the Grading of Recommendations Assessment, Development, and Evaluation (GRADE) system of rating quality of evidence [41].
*Composition not stated. According to a paper from another group, Procapil™ contains undisclosed amounts of oleanolic acid (extracted from olive leaves), apigenin (a flavonoid extracted from citrus peel) and glycine-histidine-lysine peptides, and biotinyl-GHK formed from biotin (vitamin H) [6].

References for Table S2:

1. Welzel, J.; Wolff, H.H; Gehring, W. Reduction of telogen rate and increase of hair density in androgenetic alopecia by a cosmetic product: Results of a randomized, prospective, vehicle-controlled double-blind study in men. *J. Cosmet. Dermatol*. **2022**, *21*, 1057-1064. https://doi.org/10.1111/jocd.14158
2. Chen, D.; Yu, F.; Wang, C.; Chen, H.; Tan, J.; Shi, Q.; He, X.; Liu, X.; Wang, F.; Zhao, H. Anti-hair loss effect of a shampoo containing caffeine and adenosine. *J. Cosmet. Dermatol*. **2024**, *00*, 1-7. https://doi.org/10.1111/jocd.16347
3. Merja, A.; Patel, N.; Patel, M.; Patnaik, S.; Ahmed, A.; Maulekhi, S. Safety and efficacy of REGENDIL™ infused hair growth promoting product in adult human subject having hair fall complaints (alopecia). *J. Cosmet. Dermatol.* **2023**, *23*, 938-948. https://doi.org/10.1111/jocd.16084

4. Rapaport, J.; Sadgrove, N.J.; Arruda, S.; Swearingen, A.; Abidi, Z.; Sadick, N. Real World, Open-Label Study of the Efficacy and Safety of a Nowel Serum in Androgenetic Alopecia. *J. Drugs Dermatol.* **2023**, *22*, 559-564. https://doi.org/10.1111/jocd.16347
5. Samadi, A.; Rokhsat, E.; Saffarian, Z.; Goudarzi, M.M.; Kardeh, S.; Nasrollahi, S.A.; Firooz, A. Assessment of the efficacy and tolerability of a topical formulation containing caffeine and Procapil 3% for improvement of male pattern hair loss. *J Cosmet Dermatol* **2024**, *23*, 1492-1494, https://doi.org/10.1111/jocd.16102.
6. Karaca, N.; Akpolat, N.D. A Comparative Study between Topical 5% Minoxidil and Topical "Redensyl, Capixyl, and Procapil"Combination in Men with Androgenetic Alopecia. *J Cosmetol Trichol* **2019**, *5*, 140, doi:10.4172/2471-9323.1000140
